# History of the Royal Academy of Sciences for the Year 1779; with the Mathematical and Philosophical Memoirs for That Year, Taken from the Register of the Academy

**Published:** 1784

**Authors:** 


### LONDON MEDICAL JOURNAL,

### For OCT. NOVEMBER, and DECEMBER, 1784.

### SECTION I.

### BOOKS.

I. *Histoire de l'Academie Royale des Sciences. Année* 1779. *Avec les Memoires de Mathematique et de Physique pour la même année, tirés des Registres de cette Academie.* i. e. *History of the Royal Academy of Sciences for the year* 1779; *with the Mathematical and Philosophical Memoirs for that year, taken from the Register of the Academy.* 4to. Paris, 1782. 653 pages, with 16 copper plates.

IN the historical part of this volume, we find the eulogy of Joseph de Jussieu, brother of Anthony and Bernard de Jussieu, names of great celebrity in the botanic world. Joseph, who was the youngest of sixteen children, was born at Lyons, Sept. 3, 1704. In his youth, he attached himſelf with great eagerneſs to the mathematics; but the reputation two of his brothers had acquired in botany, ſoon induced him to purſue the ſame career, and to devote himſelf to the profeſſion of phyſic. In 1735, he was appointed, as botaniſt, to accompany the French academicians to Peru, where he made thoſe obſervations on the different ſpecies of bark which we have already had occaſion to mention (vol. iv. p. 305.) When his aſtronomical friends had accompliſhed the object of their miſſion, M. de Juſſieu, inſtead of returning with them to Europe, choſe rather to make a longer ſtay in Peru, with a view to explore its natural hiſtory. It has been ſaid alſo, that an epidemic diſeaſe happening to prevail at that time, the inhabitants, who venerated his medical talents, inſiſted on his not quitting the country till the diſeaſe was over; and even threatened to puniſh any one who ſhould be acceſſary to his eſcape. Among his papers have been found ſome notes, relative to this and other epidemics, and likewiſe concerning the ſmall-pox, as it appears in Peru. He was prevented by ill health, and his profeſſional engagements, from ſetting out on his intended travels into the interior parts of Peru till 1747.

His

His biographer gives an account of the ſingular hardſhips and dangers he had to encounter in thoſe travels, in which he employed three years, and in the courſe of which he made many uſeful diſcoveries in natural hiſtory.

In 1750, he returned to Potoſi, which he had quitted three years before, and here he paſſed ſome years in drawing a map of the province, examining the mines, aſſiſting in ſeveral public works, and practiſing phyſic. In 1755, he removed to Lima, where he continued many years; but at length his health began to decline, and he became ſubject to frequent vertigo, which ended in an almoſt total loſs of memory. His friends at Lima commiſerating his ſituation, procured him a paſſage to Europe, and he arrived at Paris in 1771, after having been abſent thirty-ſix years. He knew that one of his brothers, Bernard de Juſſieu, was ſtill living; but this was almoſt the only thing he was ſenſible of, ſo much were his faculties impaired. He continued in this ſtate of imbecility till his death, which happened on the 11th of April, 1779.

It is mentioned as a remarkable circumſtance, that although he was for thirty-ſix years a member of the academy, (having been elected in 1743) he was never present at one of its meetings; the state of his intellects, after his return to his native country, having been such as disabled him from appearing in public.—His nephew, Dr. Anthony de Jussieu, means, we are told, to publish some account of his travels, collected from the papers he has left behind him.

In the second part of the volume, we meet with the following memoirs:

1. *Of the structure of the organs which serve in the formation of the voice, considered in man, and in the different classes of animals, and compared with each other. By M.* Vicq D'Azyr.—We have here a very accurate description of the organ of voice as observed in man, in the ape, in quadrupeds of different kinds, in birds, and in reptiles. Several species of ape, it seems, are furnished with membranous bags, which communicate with the larynx, and by being alternately filled with and emptied of air, serve to form the cry of those animals. The structure of this bag in the Orang-Outang has lately been described by Professor Camper, in the *Philos. Trans.* for 1779. In two species of monkey, (*ouraine* and *alouate* of Buffon; *preacher monkey*, and *royal monkey* of Pennant) which, which, on account of the ſtrength of their voice, have been called *ſinges-hurleurs*, or *howling monkies*, this reſervoir is found to be a bony pouch. In the larynx of the cat, our author has obſerved two membranes, which have eſcaped the notice of Severinus and Blaſius, and which vibrate on blowing into the trachea, and produce a noiſe ſimilar to the purring of a cat. In the hog, the glottis, we are told, has ſeveral cavities covered by a muſcle; and in the aſs and the mule, the thyroid cartilage is furniſhed with a cavity, covered with a membrane, ſo as to form a kind of drum. The air which is alternately forced into and out of theſe cavities, increaſes the voice of theſe animals, and produces the ſounds which characterize them. The organ of voice in birds is conſtructed very differently from that of quadrupeds; they have no epiglottis, but their glottis has the faculty of opening and ſhutting. This is the only part of the organ that is at the upper part of the neck; the reſt is at the lower part, above the bifurcation of the bronchiæ, the membranes of which are capable of vibration, and ſeem to anſwer the ſame purpoſe as the membranes which in man are called vocal chords. In ſome ſpecies, as in the ſwan, the trachea ſinks into into the ſternum; in others, it extends outwards on each ſide of that bone. This diſpoſition our author ſuppoſes to be analogous to the bony pouches, and large cavities obſervable in certain quadrupeds; and of courſe he conſiders it as intended only to give ſtrength to the voice, eſpecially as he has found no ſuch apparatus in thoſe ſpecies of birds whoſe notes are varied and agreeable. In general, he has obſerved, that the organ of voice, like that of hearing, is moſt perfect in thoſe animals where its ſtructure is the moſt ſimple. In frogs, the vocal chords are found, but no epiglottis. In reptiles, the glottis ſeems to form the whole of the organ, and their voice, it is obſerved, is confined to hiſſing.

The reſults of our author's obſervations are, 1. That the glottis ſeems to be of no uſe in forming ſounds; 2. that the inferior membranes of the larynx in man and in quadrupeds, and the elaſtic membranes of the bronchiæ in birds, are the true organ of voice, as they are the only parts ſuſceptible of vibration; 3. that the bony pouches, and cavities of the trachea, which exiſt in many ſpecies of animals, ſerve only to increaſe the intenſity of the ſound.— Theſe concluſions, however, he does not mean to to offer as decisive, as he intends to prosecute his inquiries on this subject. His descriptions in the present paper are illustrated by a great number of elegant figures in seven plates.

II. *Analysis of the yellow bolar earth of Berry. By* M. Sage.—It is from this earth that is procured the red colouring substance, known by the name of Prussian or English red. The Dutch purchase it in Berry, at the rate of thirty-eight or forty sols per quintal; and after giving it a red colour, sell it again at different prices, from twenty-five to forty-eight livres per quintal, according to the goodness of the colour. The profit of the Dutch on this article has long been known; but their mode of preparing it has been kept secret. M. Sage has discovered that it consists simply in colouring it. A quintal of this earth, submitted to chemical analysis, yielded of acidulous water ten pounds, of calx of iron forty pounds, and of a colourless argillaceous earth fifty pounds.

III. *Case of a strangulated intestine. By* M. Bordenave.—The subject of this history was a man, forty-five years old, who was attacked with a violent cholic, which terminated fatally in eighteen days. On dissection, the ileon was found adhering to and strangulated in a pouch formed formed in the peritonæum. Our author conjectures, that this diſtenſion of the peritonæum originated from ſome violent exertion, and that ſome of the common cauſes of inflammation produced the ſtrangulation of the inteſtine and death.

IV. *Three Memoirs on the means of diſſolving platina in the nitrous acid. By* M. Tillet.—From theſe inquiries, we learn that platina, which, on account of its ſpecific gravity, and other qualities, in ſome reſpects approaches to gold, is, however, not to be conſidered as a perfect metal, as the nitrous acid ſeparates from it a black powder, which has no metallic property, and which has hitherto appeared to be irreducible.

V. *On the population of Paris, and the provinces of France, ſince the beginning of the preſent century. By* M. Morand.—It is a queſtion with philoſophers and politicians, whether Europe is more populous now than in the time of the Roman republic or under the Emperors. Monteſqieu and Wallace contend that the human ſpecies have diminiſhed in number ſince the times we call *ancient*; but this opinion is oppoſed by Hume and Voltaire. The author of the paper now before us confines his inquiries to to France; and after a careful examination of the different authors who have written on the population of that country, he concludes, that within the laſt forty years, the number of its inhabitants has conſiderably increaſed. In 1682, according to an account taken by order of M. Colbert, Paris contained 720,000 inhabitants. In 1760, the number, according to the Abbé Expilly, was reduced to 600,000, or to 558,000, according to M. de Buffon. The population is therefore conſiderably diminiſhed ſince the time of M. Colbert; but as, during the laſt forty years, the number of births has conſtantly ſurpaſſed that of deaths, our author contends that the population of Paris is now again on the increaſe. Among other proofs that the population of the provinces is likewiſe on the increaſe, he quotes the obſervations of M. Maiſſance, who, from accurate inquiries in 128 pariſhes of Auvergne, Lyons, and Rouen, found that in the ſpace of ſixty-two years, the population had increaſed more than 1 11th. The ſame writer, we are told, confirms the remark of M. Malouin, that the months of July, May, June, and Auguſt are the moſt frequent dates of conception; and November, March, April,

 and and October, the least so, in the order in which they stand.

VII. *On the Vitriol of Mercury.* *By* the same.—Some chemists have asserted, that if we attempt to sublime vitriol of mercury, this substance will be decomposed instead of being sublimed; and that the metal, detached from the acid, will come over in the form of running mercury. But M. Cornette, who has sublimed a great quantity of this vitriol, asserts that only a very small portion of the mercury is separated from the acid, and that the mercury, so detached, is not in a fluid state, but in the form of a gray powder. He observes also, that when calx of mercury is employed instead of fluid mercury, mercury, in making the vitriol of mercury, the latter will be ſublimed entire.

VIII. *On the decompoſition of ſeveral vitriolic and nitrous ſalts with a metallic baſis, by means of the marine acid. By* the ſame. — The author's view, in this paper, is to examine the action of the marine acid on the vitriolic and nitrous ſalts with a metallic baſis. In the preceding volume (*ſee our 4th vol. p.* 28,) he had ſhewn, that this acid decompoſes the vitriolic and nitrous ſalts with baſes of fixed and volatile alkali; and he now proves that its action is the ſame on the ſalts mentioned in the title of the preſent paper. This property was already known with regard to ſilver, lead, mercury, and regulus of antimony; but M. Cornette here extends it to copper, iron, zinc, and cobalt.

IX. *Of the different Salts obtained by lixiviation from the aſhes of Tamariſk, procured in different places. By* the ſame.——It has long been obſerved, that the aſhes of this plant (*Tamariſcus Germanica* LINN.) yield no fixed alkali, but only a vitriolic ſalt; but we here learn, for the firſt time, that this vitriolic ſalt will be almoſt entirely Glauber's ſalt, if the plant grows near the ſea; and that in proportion as the tamariſk grows remote from the ſea,

 this this ſalt will be more and more a ſalt of vitriolated tartar, ſo that in very inland ſituations it will afford no veſtige of Glauber's ſalt. The aſhes of this plant are uſed for decompoſing the mother water of ſaltpetre, and for procuring nitre. The place of its growth, therefore, is not matter of indifference. On the borders of the ſea, M. Cornette obſerves, it will afford moſt quadrangular nitre; while, on the contrary, in inland ſituations, it will yield only true ſaltpetre.

x. *Botanico-meteorological obſervations made in the Caſtle of Denainvilliers, near Pithiviers, in Gatinois, in the year* 1778, *by* M. Du Hamel.

